# High Serum IgE is Associated with Risk of Severe Exacerbations Among Non-Eosinophilic Bronchiectasis

**DOI:** 10.1007/s00408-026-00874-2

**Published:** 2026-04-27

**Authors:** Ting-Wei Kao, Ya-Hui Wang, Chia-Ling Chang, Chau-Chyun Sheu, Ping-Huai Wang, Meng-Heng Hsieh, Wu-Huei Hsu, Ming-Tsung Chen, Wei-Fan Ou, Yu-Feng Wei, Tsung-Ming Yang, Chou-Chin Lan, Cheng-Yi Wang, Chih-Bin Lin, Ming-Shian Lin, Yao-Tung Wang, Ching-Hsiung Lin, Shih-Feng Liu, Meng-Hsuan Cheng, Yen-Fu Chen, Wen-Chien Cheng, Chung-Kan Peng, Ming-Cheng Chan, Ching-Yi Chen, Lun-Yu Jao, Chi-Jui Chen, Shih-Pin Chen, Yi-Hsuan Tsai, Shih-Lung Cheng, Horng-Chyuan Lin, Jung-Yien Chien, Hao-Chien Wang, Taiwan Bronchiectasis Research Collaboration (TBARC)

**Affiliations:** 1 Department of Internal Medicine, National Taiwan University Hospital, National Taiwan University College of Medicine, Taipei, Taiwan; 2 Medical Research Center, Cardinal Tien Hospital and School of Medicine, College of Medicine, Fu Jen Catholic University, New Taipei City, Taiwan; 3 Department of Internal Medicine, National Taiwan University Hospital Hsin-Chu Branch, HsinChu, Taiwan; 4 Division of Pulmonary and Critical Care Medicine, Department of Internal Medicine, Kaohsiung Medical University Hospital, Kaohsiung, Taiwan; 5 Department of Internal Medicine, School of Medicine, College of Medicine, Kaohsiung Medical University, Kaohsiung, Taiwan; 6 Division of Thoracic Medicine, Far Eastern Memorial Hospital, New Taipei City, Taiwan; 7 Department of Thoracic Medicine, Chang Gung Memorial Hospital, Linkou, Taoyuan, Taiwan; 8 College of Medicine, Chang Gung University, Taoyuan, Taiwan; 9 Division of Pulmonary and Critical Care Medicine, Department of Internal Medicine, China Medical University Hospital, Taichung, Taiwan; 10 Critical Medical Center, China Medical University Hospital, Taichung, Taiwan; 11 School of Medicine, College of Medicine, China Medical University, Taichung, Taiwan; 12 Division of Pulmonary and Critical Care Medicine, Department of Internal Medicine, Tri-Service General Hospital, National Defense Medical University, Taipei, Taiwan; 13 Division of Chest Medicine, Department of Internal Medicine, Taichung Veterans General Hospital, Taichung, Taiwan; 14 School of Medicine for International Students, College of Medicine, I-Shou University, Kaohsiung, Taiwan; 15 Department of Internal Medicine, E-Da Cancer Hospital, I-Shou University, Kaohsiung, Taiwan; 16 Division of Pulmonary and Critical Care Medicine, Chiayi Chang Gung Memorial Hospital, Chiayi, Taiwan; 17 Division of Pulmonary Medicine, Department of Internal Medicine, Taipei Tzu Chi Hospital, Buddhist Tzu Chi Medical Foundation, New Taipei City, Taiwan; 18 Department of Internal Medicine, Cardinal Tien Hospital and School of Medicine, College of Medicine, Fu Jen Catholic University, No. 362, Zhongzheng Road, Xindian District, New Taipei City, 23148 Taiwan; 19 Division of Pulmonary Medicine, Department of Internal Medicine, Hualien Tzu Chi Hospital, Buddhist Tzu Chi Medical Foundation, Hualien, Taiwan; 20 School of Medicine, Tzu-Chi University, Hualien, Taiwan; 21 Division of Pulmonary Medicine, Department of Internal Medicine, Chia-Yi Chrisitian Hospital, Chiayi, Taiwan; 22 Division of Pulmonary Medicine, Department of Internal Medicine, Chung Shan Medical University Hospital, Taichung, Taiwan; 23 School of Medicine, Chung Shan Medical University, Taichung, Taiwan; 24 Department of Internal Medicine, Division of Chest Medicine, Changhua Christian Hospital, Changhua, Taiwan; 25 Institute of Genomics and Bioinformatics, National Chung Hsing University, Taichung, Taiwan; 26 Ph.D. Program in Translational Medicine, National Chung Hsing University, Taichung, Taiwan; 27 Division of Pulmonary & Critical Care Medicine, Department of Internal Medicine, Kaohsiung Chang Gung Memorial Hospital, Kaohsiung, Taiwan; 28 Department of Respiratory Therapy, Kaohsiung Chang Gung Memorial Hospital, Kaohsiung, Taiwan; 29 Department of Respiratory Therapy, College of Medicine, Kaohsiung Medical University, Kaohsiung, Taiwan; 30 Department of Internal Medicine, Yunlin Branch, National Taiwan University Hospital, Douliu, Taiwan; 31 Thoracic Medicine Center, Yunlin Branch, National Taiwan University Hospital, Douliu, Taiwan; 32 Department of Medicine, Hualien Armed Forces General Hospital, Hualien, Taiwan; 33 Department of Critical Care Medicine, Taichung Veterans General Hospital, Taichung, Taiwan; 34 School of Post Baccalaureate Medicine, College of Medicine, National Chung Hsing University, Taichung, Taiwan; 35 Department of Internal Medicine, E-Da Hospital, I-Shou University, Kaohsiung, Taiwan; 36 Department of Pulmonary Medicine, Lees Clinic, Pingtung, Taiwan; 37 Department of Respiratory Therapy, Chang Gung Memorial Hospital, Linkou, Taoyuan, Taiwan; 38 Department of Medicine, National Taiwan University Cancer Center, National Taiwan University College of Medicine, Taipei, Taiwan

**Keywords:** Bronchiectasis, Immunoglobulin E, Eosinophilic, Type 2 inflammation, Exacerbation

## Abstract

**Purpose:**

Bronchiectasis has traditionally been characterized as a neutrophil-driven disease, yet emerging evidence suggested inflammatory heterogeneities. The prognostic significance of elevated serum immunoglobulin E (IgE) in patients without peripheral eosinophilia remains unclear.

**Methods:**

We conducted a multicenter prospective cohort study between 2017 and 2020 across 16 institutions in Taiwan. Individuals with bronchiectasis but without allergic bronchopulmonary aspergillosis were included. Patients were stratified by baseline absolute eosinophil count (cutoff 300 /uL) and serum IgE level (≤ 100, 100–500, > 500 IU/mL). The primary endpoint was severe exacerbations resulting in hospitalization at one year. Secondary endpoints included all-cause mortality, distribution of sputum pathogen, imaging pattern, and lung function.

**Results:**

A total of 579 individuals were enrolled. Nontuberculous mycobacteria (10.7%) and *Pseudomonas aeruginosa* (9.0%) were the most commonly isolated microorganisms in sputum. 493 patients (85.1%) were categorized as low-eosinophil bronchiectasis, and 41 (7.1%) presented serum IgE levels exceeding 500 IU/mL. The rate of hospitalization for acute exacerbation in such group was pronouncedly higher than in patients with lower IgE levels (9.8% vs. 0.9% and 2.3%; *P* = 0.009). In multivariate analysis, IgE exceeding 500 IU/mL was the strongest independent predictor of hospitalization (adjusted odds ratio, 7.38; 95% confidence interval, 2.40–22.7; *P* < 0.001). The association was particularly pronounced in female and patients with coexisting asthma. All-cause mortality did not differ significantly among IgE strata.

**Conclusion:**

Markedly elevated serum IgE independently predicted severe exacerbations resulting in hospitalization in patients with non-eosinophilic bronchiectasis, identifying a high-risk subgroup that may benefit from targeted immunomodulatory therapies.

**Electronic supplementary material:**

The online version of this article (10.1007/s00408-026-00874-2) contains supplementary material, which is available to authorized users.

## Introduction

Bronchiectasis is characterized by permanent dilation of bronchi with chronic airway inflammation and recurrent infections [1]. Although traditionally conceptualized as a predominantly neutrophil-driven disease, accumulating evidence suggests substantial inflammatory heterogeneities within bronchiectasis populations. Approximately 20–40% of patients with bronchiectasis exhibited features of type 2 immune activation, including elevated serum immunoglobulin E (IgE), blood eosinophil count, and fractional exhaled nitric oxide, supporting the epidemiological and prognostic relevance of type 2-associated endotypes in bronchiectasis [2, 3].

Blood eosinophil count and IgE both serve as pivotal biomarkers and interconnected effectors in type 2 inflammation yet reflecting distinct immunopathologic processes. Eosinophil activation and recruitment are primarily driven by interleukin-5, ultimately leading to allergic responses and tissue remodeling. In contrast, IgE production is induced by T helper 2 cells and innate lymphoid cells. Cross-linking of IgE bound to mast cells and basophils triggers rapid degranulation with release of histamine and leukotrienes upon allergen exposure [4], resulting in immediate hypersensitivity reactions and amplified local inflammation. In severe asthma, upregulation of IgE through activated interleukin-4 and interleukin-13 signaling pathways has been demonstrated contributing to eosinophilic inflammation, mucus hypersecretion, and airway remodeling [5].

Epidemiologic study documented that elevated IgE is present in at least one third of patients with bronchiectasis even without asthma or allergic bronchopulmonary aspergillosis [6], implicating the existence of a distinct type 2-high phenotype. Nevertheless, preliminary studies have focused predominantly on radiologic image manifestations. A single-center retrospective study reported that elevated IgE may be associated with a greater number of involved lung lobes and more frequent mucus plugging on high-resolution computed tomography, regardless of eosinophil level [7]. The positive correlation between IgE and the extent of bronchiectatic involvement on imaging has been observed in bronchiectasis with concomitant allergic bronchopulmonary aspergillosis [8] or chronic obstructive pulmonary disease [9] as well.

However, how these different endotypes alter symptoms, sputum pathogen distribution, lung function, and exacerbation rates remains unknown. The lack of endotyping by IgE level, both with and without concomitant eosinophilia, in relation to clinical manifestations marks a clinical unmet need. Our study aimed to determine the prognostic role of serum IgE levels in predicting adverse outcomes, particularly in individuals with non-eosinophilic bronchiectasis.

## Methods

### Study Design and Population

The multicenter prospective observational cohort study was conducted between 2017 and 2020 across 16 institutions affiliated with Taiwan Bronchiectasis Research Collaboration (TBARC). Inclusion criteria were adults older than 20 years of age with bronchiectasis [10] and at least two outpatient clinic visits. Bronchiectasis was diagnosed according to 2017 European Respiratory Society guidelines and based on clinical and radiological criteria as defined previously [11], which requires the presence of bronchial dilation on high-resolution computed tomography in conjunction with compatible clinical features including chronic cough, sputum production, or recurrent respiratory infections. Subjects with allergic bronchopulmonary aspergillosis (ABPA) were excluded due to the inherently elevated IgE levels and well-established association with exacerbations. Specifically, patients with documented ABPA diagnosis, characteristic radiologic findings as central bronchiectasis with mucoid impaction, or elevated aspergillus-specific IgE levels were excluded. All patients were evaluated by pulmonologists who excluded clinically suspected ABPA based on history, laboratory examination, and radiologic features. Asthma was diagnosed clinically according to Global Initiative for Asthma (GINA) guidelines. Asthma was not an exclusion criterion given the substantial clinical overlap between asthma and bronchiectasis and we aimed to evaluate whether IgE retained prognostic value across the heterogeneous population. Since the study location is not an endemic region for parasitic infections, elevated IgE secondary to parasitic infestation was considered rare.

### Data Collection

We systematically collected baseline demographics, clinical symptoms, medical comorbidities, microbiologic data, radiographic findings, and pulmonary function test results at enrollment. Chest high-resolution computed tomography was implemented to assess anatomic involvement and disease severity. Images were reviewed by pulmonologists and thoracic radiologists who were blinded to clinical data. The modified Reiff score (range 0–18; higher scores indicating more severe disease) was applied to quantify radiographic severity [12].

Sputum specimens were obtained during clinical stability by spontaneous expectoration or bronchial washing according to standardized protocols. The exact timing and method for sputum collection were at the discretion of treating pulmonologist. Bacterial culture was performed by inoculating specimens onto blood agar, eosin-methylene blue agar, and chocolate agar plates, followed by incubation at 35 °C in a 5% CO_2_ atmosphere. Pathogen identification was conducted using biochemical methods in conjunction with matrix-assisted laser desorption/ionization time-of-flight mass spectrometry (Bruker Biotyper MALDI-TOF MS platform). Acid-fast smear and culture for mycobacteria were managed according to previously described protocol [13]. Pulmonary function testing was operated in accordance with American Thoracic Society and European Respiratory Society standards [14] and interpreted with healthy non-smoking Chinese individuals as reference population for predicted forced expiratory volume in one second and forced vital capacity [15].

### Stratification and Outcomes

Subjects were initially categorized by baseline absolute eosinophil count with threshold at 300 /uL defining high- or low-eosinophil bronchiectasis. Subsequently, the cohort was stratified into three groups by baseline serum IgE: 100 IU/mL or less, 100–500 IU/mL, and greater than 500 IU/mL. These cutoffs were derived from previous guidelines and cohort studies regarding allergic diseases [16–20].

Patients were followed for one year or until death, whichever occurred first. The primary endpoints were any hospitalization for severe acute exacerbation. Acute exacerbation was defined according to established criteria as deterioration in at least three key symptoms lasting more than 48 h [21]. Such endpoint was refined during peer-review process from the originally proposed all-cause mortality, which was designated as a secondary endpoint to provide exploratory data on long-term prognosis, because of power limitations in detecting differences within one year period. Additional secondary endpoints included pathogen distribution in sputum, image pattern, and lung function.

### Statistical Analysis

Categorical variables were presented as numbers and percentages. Continuous variables were expressed as means and standard deviations. Comparisons of categorical and continuous variables among the three IgE strata were performed using the chi-square test and one-way analysis of variance, respectively. Multivariate logistic regression with stepwise forward selection was applied to identify independent risk factors after adjustment for established confounders, including age, sex, body-mass index, smoking status, asthma, chronic obstructive pulmonary disease, inhaled corticosteroid and long-acting beta-adrenoceptor agonists, and eosinophilia. Variance inflation factors were evaluated to assess potential collinearity among covariates in multivariate models, and the value less than 3.0 indicated acceptable levels of collinearity. The association was expressed as odds ratio (OR) with 95% confidence interval (CI). Prespecified sensitivity analyses stratified by age, sex, asthma, chronic obstructive pulmonary disease, presence of tree-in-bud pattern, and eosinophilia were conducted to investigate the robustness of the association between IgE greater than 500 IU/mL and outcomes. A two-tailed *P* value of less than 0.05 was considered statistically significant. All analyses were performed using SPSS software packages, version 19 (IBM Corporation, Armonk, NY).

## Results

**Fig. 1:**
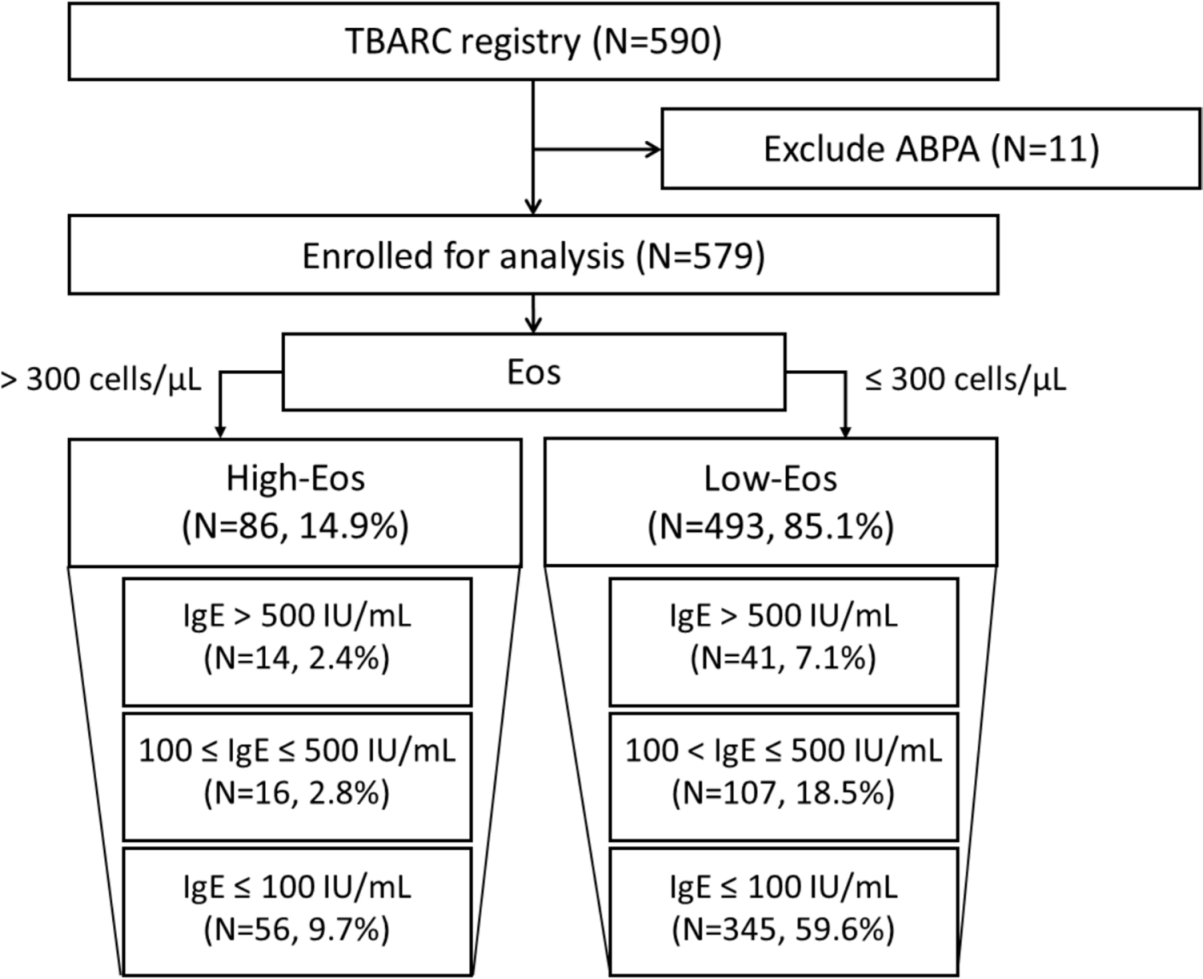
Study flow diagram. The cohort was categorized based on serum IgE level and absolute eosinophil count. ABPA, allergic bronchopulmonary aspergillosis; Eos, absolute eosinophil count; IgE, immunoglobulin E

A total of 590 individuals were initially screened, and 579 subjects were included in the analysis (Fig. 1). The mean age of the entire cohort was 66.2 years, with 55.6% female. A total of 493 patients (85.1%) were categorized as low-eosinophil, of whom 41 (7.1%) exhibited serum IgE levels exceeding 500 IU/mL. The distribution of absolute eosinophil counts in respective to IgE level was summarized **(Supplementary Fig. 1)**.

In the low-eosinophil group, baseline demographic characteristics and clinical features were otherwise comparable (Table 1) except that patients with IgE exceeding 500 IU/mL were more likely to be receiving inhaled corticosteroid with long-acting beta-agonist combination therapy (43.9%; *P* = 0.003) and biologic agents (12.2%; *P* < 0.001) compared with those in lower IgE strata. In the high-eosinophil cohort, elevated IgE was significantly associated with greater prevalence of hemoptysis (50% in the group with IgE > 500 IU/mL vs. 6.3% in the group with IgE of 100 to 500 IU/mL and 12.5% in the group with IgE < 100 IU/mL; *P* = 0.002) and allergic rhinitis (71.4% vs. 31.3% and 37.5%; *P* = 0.045). Smokers demonstrated significantly greater eosinophil count (189.5 ± 0.49 vs. 173.0 ± 0.50 /uL; *P* < 0.001) but similar IgE level (156.3 ± 301.3 vs. 168.9 ± 339.8 IU/mL; *P* = 0.67) as compared with non-smokers **(Supplementary Table 1)**.

**Table 1:** Clinical characteristics stratified by IgE level in high-eosinophil and low-eosinophil groups

	Entire cohort (N = 579)	High-eosinophil group					Low-eosinophil group				
		All (N = 86)	IgE ≤ 100 IU/mL (N = 56)	100 < IgE ≤ 500 IU/mL (N = 16)	IgE > 500 IU/mL (N = 14)	*P*	All (N = 493)	IgE ≤ 100 IU/mL (N = 345)	100 < IgE ≤ 500 IU/mL (N = 107)	IgE > 500 IU/mL (N = 41)	*P*
Age (year)	66.2 ± 11.9	63.1 ± 12.6	61.7 ± 13.8	69.4 ± 10.4	61.4 ± 7.7	0.09	66.8 ± 11.7	67.0 ± 11.3	66.7 ± 13.3	65.2 ± 10.9	0.63
Female	328 (56.6)	39 (45.3)	26 (46.4)	4 (25.0)	9 (64.3)	0.09	289 (58.6)	203 (58.8)	64 (59.8)	22 (53.7)	0.78
Body mass index (kg/m^2^)	22.5 ± 4.1	23.2 ± 3.9	23.8 ± 3.9	22.6 ± 3.4	21.3 ± 3.9	0.08	22.3 ± 4.09	22.5 ± 4.20	22.2 ± 3.67	22.0 ± 4.23	0.49
Smoker	171 (29.5)	34 (39.5)	21 (37.5)	7 (43.8)	6 (42.9)	0.87	137 (27.8)	100 (29.0)	27 (25.2)	10 (24.4)	0.66
*Symptoms*											
Hemoptysis	116 (20.0)	15 (17.4)	7 (12.5)	1 (6.3)	7 (50.0)	**0.002**	101 (20.5)	67 (19.4)	24 (22.4)	10 (24.4)	0.65
Dyspnea	305 (52.7)	48 (55.8)	34 (60.7)	9 (56.3)	5 (35.7)	0.24	257 (52.1)	178 (51.6)	58 (54.2)	21 (51.2)	0.89
Cough	476 (82.2)	63 (73.3)	41 (73.2)	11 (68.8)	11 (78.6)	0.83	413 (83.8)	284 (82.3)	92 (86.0)	37 (90.2)	0.34
Sputum	436 (75.4)	66 (76.7)	43 (76.8)	12 (75.0)	11 (78.6)	0.97	370 (75.1)	258 (74.8)	82 (76.6)	30 (73.2)	0.90
*Comorbidities*											
Asthma	212 (36.6)	36 (41.9)	21 (37.5)	8 (50.0)	7 (50.0)	0.53	176 (35.7)	119 (34.5)	42 (39.3)	15 (36.6)	0.66
Allergic rhinitis	222 (38.3)	36 (41.9)	21 (37.5)	5 (31.3)	10 (71.4)	**0.045**	186 (37.7)	129 (37.4)	43 (40.2)	14 (34.1)	0.77
COPD	257 (44.4)	33 (38.4)	20 (35.7)	9 (56.3)	4 (28.6)	0.24	224 (45.4)	157 (45.5)	50 (46.7)	17 (41.5)	0.85
History of pneumonia	207 (35.8)	30 (34.9)	19 (33.9)	5 (31.3)	6 (42.9)	0.78	177 (35.9)	126 (36.5)	35 (32.7)	16 (39.0)	0.70
History of tuberculosis	101 (17.4)	15 (17.4)	7 (12.5)	4 (25.0)	4 (28.6)	0.25	86 (17.4)	56 (16.2)	24 (22.4)	6 (14.6)	0.30
Hypertension	208 (35.9)	33 (38.4)	19 (33.9)	10 (62.5)	4 (28.6)	0.08	175 (35.5)	122 (35.4)	41 (38.3)	12 (29.3)	0.59
Diabetes mellitus	92 (15.9)	14 (16.3)	11 (19.6)	1 (6.3)	2 (14.3)	0.43	78 (15.8)	57 (16.5)	16 (15.0)	5 (12.2)	0.74
Cardiovascular disease	153 (26.4)	23 (26.7)	16 (28.6)	4 (25.0)	3 (21.4)	0.85	130 (26.4)	91 (26.4)	29 (27.1)	10 (24.4)	0.95
Chronic kidney disease	48 (8.3)	9 (10.5)	5 (8.9)	2 (12.5)	2 (14.3)	0.81	39 (7.9)	28 (8.1)	9 (8.4)	2 (4.9)	0.75
Solid cancer	77 (13.3)	15 (17.4)	7 (12.5)	3 (18.8)	5 (35.7)	0.12	62 (12.6)	44 (12.8)	9 (8.4)	9 (22.0)	0.08
GERD	122 (21.1)	15 (17.4)	7 (12.5)	5 (32.3)	3 (21.4)	0.20	107 (21.7)	74 (21.4)	26 (24.3)	7 (17.1)	0.62
Autoimmune disease	20 (3.5)	2 (2.3)	1 (1.8)	1 (6.3)	0	0.48	18 (3.7)	14 (4.1)	4 (3.7)	0	0.42
*Therapeutics*											
O_2_ dependence	5 (0.9)	3 (3.5)	2 (3.6)	0	1 (7.1)	0.57	2 (0.4)	2 (0.6)	0	0	0.65
NIPPV dependence	4 (0.7)	2 (2.3)	1 (1.8)	0	1 (7.1)	0.39	2 (0.4)	2 (0.6)	0	0	0.65
ICS/LABA	165 (28.5)	30 (34.9)	18 (32.1)	6 (37.5)	6 (42.9)	0.73	135 (27.4)	80 (23.2)	37 (34.6)	18 (43.9)	0.003
Dual bronchodilator	106 (18.3)	22 (25.6)	13 (23.2)	4 (25.0)	5 (35.7)	0.63	84 (17.0)	63 (18.3)	18 (16.8)	3 (7.3)	0.21
Triple therapy	12 (2.1)	3 (3.5)	2 (3.6)	1 (6.3)	0	0.65	9 (1.8)	5 (1.4)	4 (3.7)	0	0.20
Biologics	7 (1.2)	0				NA	7 (1.4)	1 (0.3)	1 (0.9)	5 (12.2)	** < 0.001**

IgE, immunoglobulin E; COPD, chronic obstructive pulmonary disease; GERD, gastroesophageal reflux disease; NIPPV, non-invasive positive pressure ventilation; ICS, inhaled corticosteroid; LABA, long-acting beta-adrenoceptor agonists. Bold texts indicated statistical significance.

**Fig. 2:**
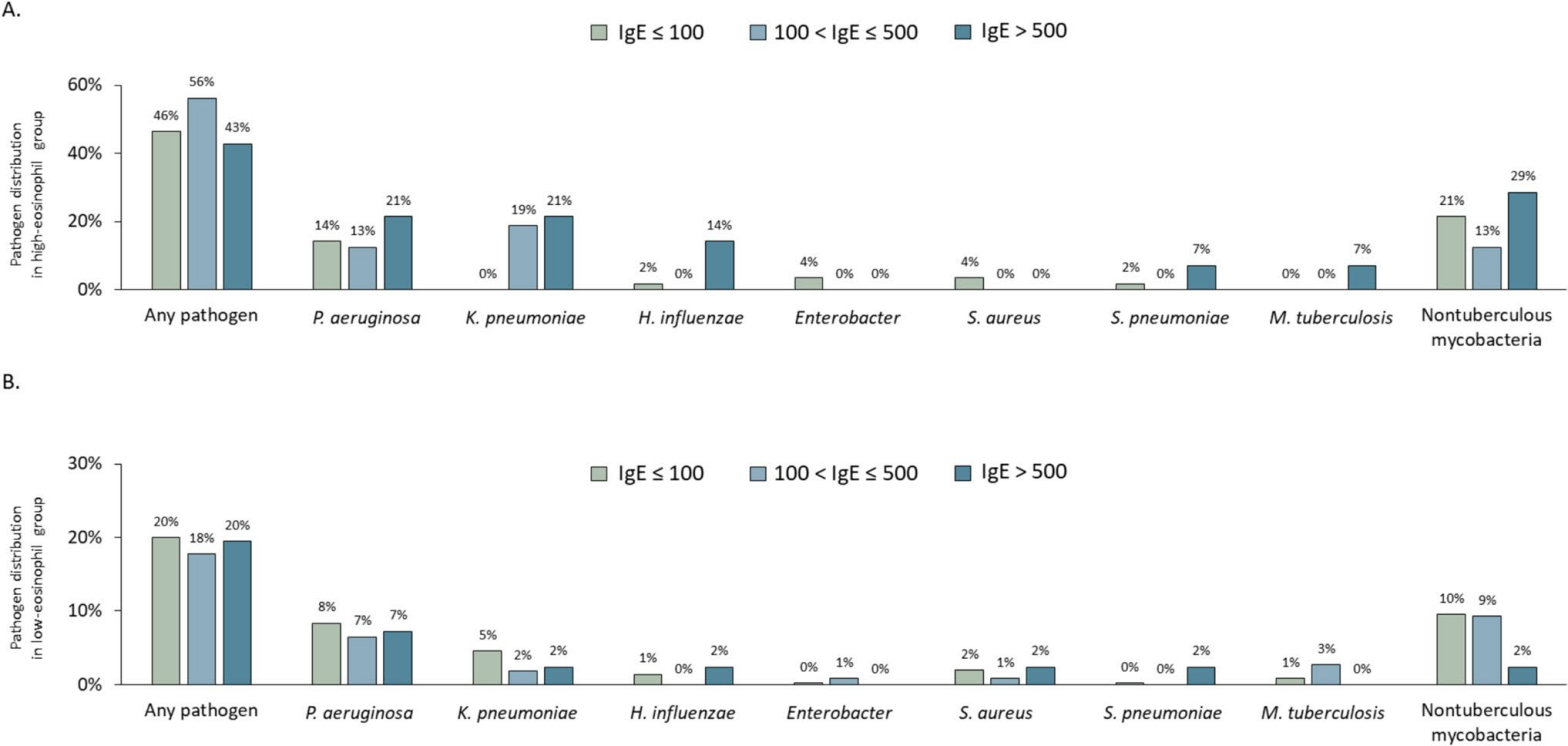
Sputum pathogen distribution across IgE and absolute eosinophil count strata. Pathogen prevalence stratified by IgE levels (100 and 500 IU/mL) in **A** high-eosinophil and **B** low-eosinophil group. IgE, immunoglobulin E

During the follow-up period, 137 patients (23.7%) had at least one pathogen identified from respiratory specimens, of which 46 patients (33.6%) were obtained by bronchoscopic washing. The detailed distribution of individual pathogens was illustrated (Fig. 2). Nontuberculous mycobacteria (62 patients, 10.7%) and *Pseudomonas aeruginosa* (52 patients, 9.0%) were the most frequently isolated microorganisms in the entire cohort and in both the high- and low-eosinophil subgroups. Overall, IgE greater than 500 IU/mL was associated with higher prevalence of *Haemophilus influenzae* (5.5%; *P* = 0.03) and *Streptococcus pneumoniae* (3.6%; *P* = 0.02) compared with other IgE strata. Among patients with high-eosinophil bronchiectasis, *Klebsiella pneumoniae* was identified exclusively in those with IgE exceeding 100 IU/mL (*P* = 0.002). In the low-eosinophil cohort, the distribution of pathogens was analogous across the three IgE strata. The associations between specific bacterial species and IgE levels were inconsistent between high- and low-eosinophil subgroups, precluding definitive conclusions about pathogen-specific relationships with type 2 inflammation.

Results of pulmonary function testing demonstrated no remarkable differences among IgE strata in either the high- or low-eosinophil populations **(Supplementary Table 2)**. Similarly, radiographic features, including lobar distribution, presence of tree-in-bud opacities, number of lobes involved, and modified Reiff scores, were equivalent across IgE groups **(Supplementary Fig. 2)**.

**Fig. 3:**
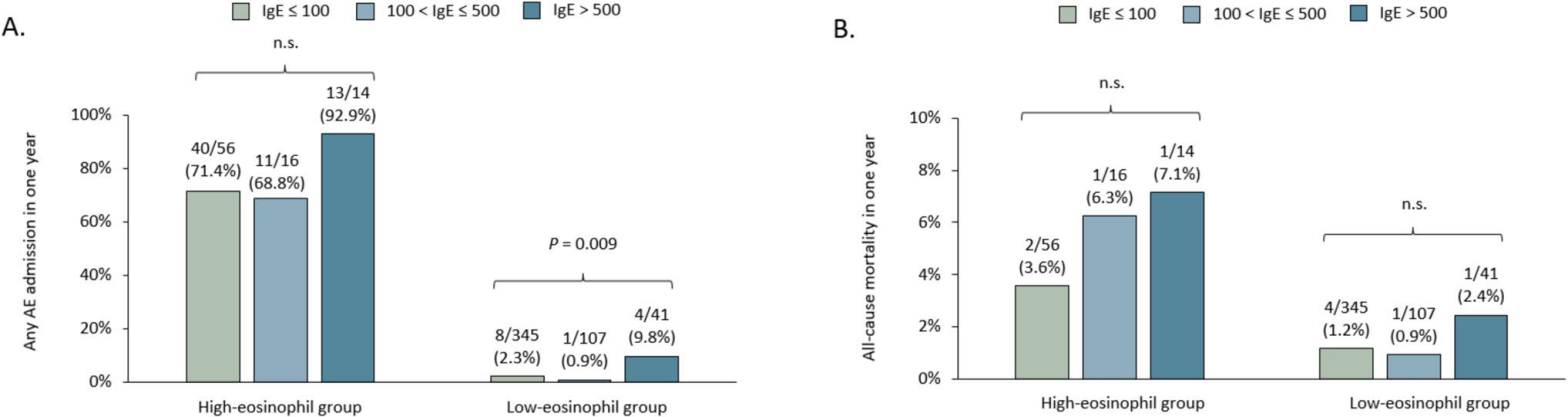
Outcomes in eosinophilic and non-Eosinophilic cohorts stratified by IgE level. Shown are the percentage of **A** AE resulting in hospitalization and **B** all-cause mortality in eosinophilic and non-eosinophilic cohort. AE, acute exacerbation; IgE, immunoglobulin; n.s.: not significant

Within the high-eosinophil cohort, the incidence of primary endpoints was notably higher overall but did not differ significantly among IgE strata. In contrast, among patients with low-eosinophil bronchiectasis, those with IgE exceeding 500 IU/mL presented a substantially higher rate of hospitalization for acute exacerbation within one year compared with those in lower IgE strata (9.8% vs. 0.9% in the intermediate-IgE group and 2.3% in the low-IgE group). As for the secondary endpoint of all-cause mortality, no statistically significant association was observed between IgE levels. In the low-eosinophil cohort, mortality rates were 2.4% for IgE > 500 IU/mL, 0.9% for IgE 100–500 IU/mL, and 1.2% for IgE < 100 IU/mL (*P* = 0.75). In the high-eosinophil cohort, corresponding mortality rates were 7.1%, 6.3%, and 3.6% across the three IgE strata (*P* = 0.81) (Fig. 3). The odds ratio of IgE > 500 IU/mL was 1.77 (95% CI, 0.17–18.4) and 2.30 (95% CI, 0.26–20.2) in the low- and high-eosinophil subgroup, respectively **(Supplementary Table 3)**.

Furthermore, univariate logistic regression identified chronic obstructive pulmonary disease (OR, 1.67; 95% CI, 1.01–2.77; *P* = 0.046), absolute eosinophil count (OR, 1.02; 95% CI, 1.01–1.02; *P* < 0.001), and IgE greater than 500 IU/mL (OR, 3.20; 95% CI, 1.69–6.09; *P* < 0.001) as potential risk factors for hospitalization due to acute exacerbation. In multivariate analysis, IgE exceeding 500 IU/mL emerged as the strongest independent predictor (adjusted OR, 7.38; 95% CI, 2.40–222.7; *P* < 0.001), along with age (OR, 1.07; 95% CI, 1.03–1.11; *P* = 0.001) and absolute eosinophil count (OR, 1.02; 95% CI, 1.02–1.03; *P* < 0.001) (Table 2). However, the small magnitude of these per-unit odd ratios might suggest limited clinical relevance. IgE > 500 IU/mL, in contrast, demonstrated both statistical significance and clinically meaningful effect size, yet the relatively small number of patients in such subgroup resulted in wide confidence interval and therein reduced prevision of such estimate. The variance inflation factors of IgE level to eosinophil count or presence of asthma were both 1.0, implying no substantial multi-collinearity among covariates in our cohort despite the biological relationships between among these covariates. The results remained similar after excluding patients administered with biologics as well **(Supplementary Table 4)**.

**Table 2:** Univariate and multivariate logistic regression analysis for severe acute exacerbation requiring admission in one year

	Univariate analysis		Multivariate analysis	
	Odds ratio	*P*	Odds ratio	*P*
Age (age)	0.992 (0.973–1.012)	0.45	1.072 (1.028–1.118)	**0.001**
Male	1.404 (0.868–2.270)	0.17	0.573 (0.163–2.019)	0.39
Body mass index (kg/m^2^)	1.031 (0.972–1.094)	0.31	0.928 (0.825–1.044)	0.21
Smoking	1.342 (0.809–2.228)	0.25	0.814 (0.224–2.966)	0.76
Asthma	1.353 (0.832–2.202)	0.22	1.312 (0.537–3.210)	0.55
Chronic obstructive pulmonary disease	1.672 (1.010–2.767)	**0.046**	1.895 (0.701–5.119)	0.21
ICS/LABA	1.522 (0.920–2.520)	0.10	2.398 (0.974–5.901)	0.06
Absolute eosinophil count (/uL)	1.017 (1.013–1.021)	**<0.001**	1.022 (1.016–1.027)	**<0.001**
Immunoglobulin E > 500 IU/mL	3.203 (1.685–6.087)	**<0.001**	7.377 (2.398–22.70)	**<0.001**

ICS, inhaled corticosteroid; LABA, long-acting beta-adrenoceptor agonists. Bold texts indicated statistical significance.

**Fig. 4:**
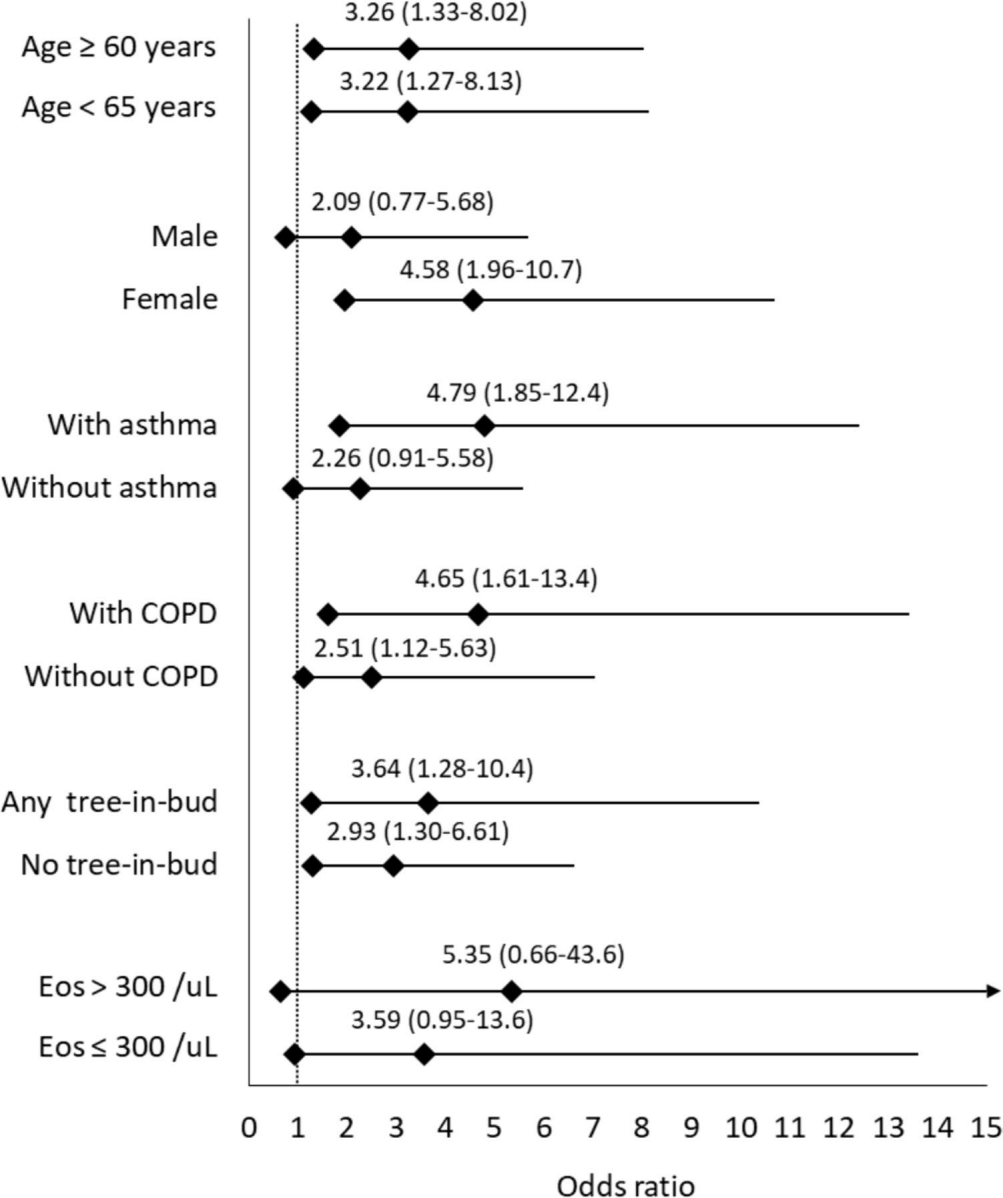
Sensitivity analyses for immunoglobulin E > 500 IU/mL and acute exacerbation resulting in hospitalization in one year. Shown are odds ratios with 95% confidence intervals across different patient subgroups. COPD, chronic obstructive pulmonary disease; Eos, absolute eosinophil count

Sensitivity analyses additionally confirmed that the association between elevated IgE and hospitalization for exacerbation was consistent across age groups but was particularly pronounced in women (OR, 4.58; 95% CI, 1.96–10.7) and in patients with coexisting asthma (OR, 4.79; 95% CI, 1.85–12.4). The presence of eosinophilia further accentuated the effect of IgE greater than 500 IU/mL (OR, 5.35; 95% CI, 0.66–43.6) (Fig. 4).

## Discussion

The study proposed markedly elevated serum IgE as a robust independent predictor of severe exacerbations resulting in hospitalization in low-eosinophil bronchiectasis. The association persisted after adjustment for established risk factors and remained consistent across different patient subgroups. Our findings did not elucidate the established role of neutrophilic inflammation, but complemented the neutrophil-driven paradigm by reporting the epidemiological and prognostic importance of type 2 immune activation as a clinically relevant endotype in bronchiectasis.

The proportion of individuals with low-eosinophil disease in our cohort aligned with prior epidemiological data from diverse geographic regions [2, 3]. Our study extended these observations by clarifying that elevated IgE conferred clinically meaningful risk independent of blood eosinophil count. Specifically, 7.1% of non-eosinophilic patients exhibited pronouncedly elevated IgE and sustained higher rate of severe acute exacerbations compared with those with lower IgE levels. These findings pointed out that type 2-skewed endotypes may be substantially under-recognized when dependent solely on peripheral eosinophil counts for phenotypic classification. The dissociation between serum IgE and blood eosinophils in a minority of patients underscored the complexity of type 2 inflammation and the need for multidimensional biomarker assessment in bronchiectasis.

Several mechanisms may account for the association between elevated IgE and unfavorable prognosis in low-eosinophil bronchiectasis, though our observational data cannot establish causality. Type 2 cytokines, particularly interleukin-4 and interleukin-13, drive IgE class-switching in B cells and promote goblet cell metaplasia, mucus hypersecretion, and impaired mucociliary clearance [22]. These alterations created a permissive environment for microbial colonization and recurrent infections, perpetuating the cycle of inflammation and progressive airway damage characteristic of bronchiectasis. Additionally, elevated IgE may directly enhance airway inflammation through cross-linking of high-affinity IgE receptors on mast cells and basophils, triggering degranulation and release of inflammatory mediators that amplify airway edema, bronchoconstriction, and mucus production [23]. These mechanisms may be particularly relevant during acute exacerbations, when allergen or pathogen exposure could trigger massive mast cell activation in sensitized individuals.

The sources of antigenic stimulation potentiating IgE production in patients without allergic bronchopulmonary aspergillosis remained incompletely defined. Chronic exposure to environmental fungi, dust mites, or microbial antigens within damaged airways may elicit sensitization and sustained IgE responses [24]. The observed associations between certain bacterial species and IgE levels in our study were inconsistent and likely reflect the complexity of host–pathogen interactions rather than causal relationships. In addition to different timing and collection method for sputum culture, the pathogenesis of elevated IgE in bronchiectasis reflected the complex interactions among environmental exposures, genetic susceptibility, and therapeutic interventions. Host factors exemplified by genetic polymorphisms related to type 2 immune pathway, epigenetic modifications, and altered regulation of IgE synthesis may predispose individuals to such phenotype. Whereas, the inhaled or systemic corticosteroids modulating the relationship between IgE level and clinical prognosis warranted further investigations. Our cross-sectional biomarker assessment cannot distinguish whether or not elevated IgE represented a primary pathogenic driver, a secondary consequence of airway damage and antigen exposure, or an intrinsic host response pattern. Besides, the heterogeneous pathogen landscape among different IgE strata observed in our study, nevertheless, possibly only reflected parallel consequences of impaired airway defense mechanisms rather than any causative link. Alternatively, these bacterial species may contribute to sustained type 2 inflammation through disruption of epithelial barrier function, modification of airway microbiome, or adjuvant effects that enhance sensitization to environmental allergens [25]. The absence of correlation between IgE levels and lung function parameters is clinically relevant and distinguishes IgE from traditional markers of disease severity. The finding persisted even in patients with coexisting asthma, suggesting that elevated IgE identified a distinct inflammatory endotype rather than simply reflecting greater structural damage or airflow obstruction.

The strongest independent predictor in our multivariate model was IgE exceeding 500 IU/mL, which substantially outperformed age and absolute eosinophil count in predicting hospitalization for exacerbations. Given the limited number of patients with markedly elevated IgE, particularly in subgroup analyses, these findings nevertheless should be considered exploratory and hypothesis-generating. Validation in larger independent cohorts is essential before definitive clinical recommendations can be established. Notably, the predictive value of elevated IgE was particularly pronounced in women and in patients with coexisting asthma, indicating the potential interactions between airway hyperresponsiveness and type 2 inflammation in determining exacerbation susceptibility. Enhanced risk observed in patients with both bronchiectasis and asthma exemplified the considerable overlap between these conditions and suggested that shared pathophysiologic mechanisms involving type 2 inflammation may drive disease progression in both contexts. Previous studies demonstrated that asthma-bronchiectasis overlap syndrome was associated with worse clinical outcomes [26, 27], and our data revealed that immunologic profiling may further identify at-risk individuals within such population. Still, multiple subgroup and sensitivity analyses might result in type I error and thus these exploratory findings should be interpreted prudently.

Recognition of a high-risk subgroup defined by elevated IgE has important therapeutic implications. Current management of bronchiectasis focused primarily on airway clearance techniques, antimicrobial therapy for acute exacerbations and chronic infections, and anti-inflammatory strategies of uncertain efficacies [10]. Our findings endorsed that patients with markedly elevated IgE may represent a distinct endotype that could benefit from targeted immunomodulatory interventions. Inhaled corticosteroids may have differential efficacy in type 2-high bronchiectasis, as suggested by data from the European Bronchiectasis Registry [28]. In addition, emerging biologic therapies targeting type 2 pathways, including anti-interleukin-5, anti-interleukin-4, and anti-IgE agents, have shown preliminary promise in case series, although current evidence is mainly derived from vases series or studies of asthma-bronchiectasis overlap [29]. While improvements in sputum production, exacerbation frequency, and lung function have been reported, these benefits have been identified primarily in patients with eosinophilia. Large-scale trials specifically targeting non-eosinophilic bronchiectasis are lacking, and data in hyper-IgE subgroups without eosinophilia remained limited. Future studies are warranted to establish the therapeutic role of these agents in bronchiectasis subgroups characterized by type 2 inflammation but lacking peripheral eosinophilia.

Our study substantiated the necessity of comprehensive endotyping in bronchiectasis management. Routine measurement of serum IgE, in conjunction with blood eosinophil count and fractional exhaled nitric oxide, may facilitate identification of patients with type 2-skewed disease who warrant intensified monitoring and consideration for immunomodulatory therapies. Implementation of such biomarker-driven approaches may improve risk stratification, guide therapeutic decision-making, and ultimately reduce the burden of severe exacerbations.

Several limitations should be addressed. First, our study was conducted exclusively in an Asian population. Literature has delineated geographic and ethnic heterogeneities in immune endotypes of bronchiectasis [30], and generalization to other ethnicities requires further cross-ethnic investigations. Second, IgE measurements were obtained at a single time point. The longitudinal trajectory of IgE levels and their correlation with clinical outcomes were not evaluated. Dynamic changes in IgE during exacerbations or in response to therapy may provide additional prognostic implications. Third, additional type 2 biomarkers aside from IgE and eosinophils were not assessed in our population. Also, only seven patients were assessed with aspergillus specific IgE. Although subclinical ABPA cannot be entirely ruled out, we considered our findings were unlikely to be substantially affected due to absence of characteristic radiologic features and clinical presentations. Fourth, the limited sample size of patients with IgE > 500 IU/mL may have resulted in over-estimation of effect size, as reflected in the wide confidence interval for the primary outcome. In addition, both the abundance of covariates included in multivariate model relative to limited outcome events and stepwise forward selection might potential result in model overfitting. Interpretation of the effect should therefore be prudent, and validation by a greater cohort is needed. Fifth, mucus plugging was not systematically assessed on high-resolution computed tomography in our cohort. Given emerging evidence linking mucus plugging to type 2 inflammation and exacerbation risk, particularly in asthma [31], the relationship between IgE levels and mucus burden represents a pivotal area for future investigations. Fifth, individuals with markedly elevated IgE were more likely to be receiving inhaled corticosteroids and biologic agents at enrollment, introducing potential confounding by indication. While sensitivity analyses excluding patients on biologics yielded similar results, these therapies may have influenced both biomarker levels and clinical outcomes. The greater treatment intensity in such subgroup also reflected clinical recognition of high-risk features, which may not be fully captured by measured covariates. Fifth, our study only investigated severe exacerbations and mortality as endpoints. Future studies are mandated to determine additional patient-reported outcomes in bronchiectasis with different endotypes. Sixth, systematic parasitologic testing was not conducted. Although the study region is not endemic for helminthic infections, elevated IgE partially attributed to parasitosis cannot be entirely excluded. Finally, residual confounding from factors including therapeutic adherence, socioeconomic determinants, and environmental exposures cannot be entirely excluded despite comprehensive adjustment for known confounders.

In conclusion, markedly elevated serum IgE independently predicted severe exacerbations resulting in hospitalization in patients with low-eosinophil bronchiectasis. These findings supported the incorporation of IgE measurement into routine assessment of bronchiectasis to identify a high-risk subgroup that may benefit from targeted interventions. Recognition of inflammatory heterogeneities will facilitate the development of individualized treatment strategies tailored by specific endotypes and improve outcomes for bronchiectasis.

## Electronic supplementary material

Below is the link to the electronic supplementary material.

Supplementary file 1 (DOCX 19 kb)

## Data Availability

The datasets generated during and/or analyzed during the current study are available from the corresponding author on reasonable request.
