# Supplementary material for: High Serum IgE is Associated with Risk of Severe Exacerbations Among Non-Eosinophilic Bronchiectasis
Source: Lung. 2026 Apr 27;204(1):25. doi: 10.1007/s00408-026-00874-2 (PMC13121277; doi:10.1007/s00408-026-00874-2)

**High Serum IgE is Associated with Risk of Severe Exacerbations among Non-eosinophilic Bronchiectasis**

Ting-Wei Kao^1^, Ya-Hui Wang^2^, Chia-Ling Chang^3^, Chau-Chyun Sheu^4,5^, Ping-Huai Wang^6^, Meng-Heng Hsieh^7,8^, Wu-Huei Hsu^9,10,11^, Ming-Tsung Chen^12^, Wei-Fan Ou^13^, Yu-Feng Wei^14,15^, Tsung-Ming Yang^16^, Chou-Chin Lan^17^, Cheng-Yi Wang^18,†^, Chih-Bin Lin^19,20^, Ming-Shian Lin^21^, Yao-Tung Wang^22,23^, Ching-Hsiung Lin^24,25,26^, Shih-Feng Liu^27,28,29^, Meng-Hsuan Cheng^4,30^, Yen-Fu Chen^31,32^, Wen-Chien Cheng^9,11^, Chung-Kan Peng^12,33^, Ming-Cheng Chan^34,35^, Ching-Yi Chen^36^, Lun-Yu Jao^20^, Chi-Jui Chen^19^, Shih-Pin Chen^22,23^, Yi-Hsuan Tsai^27,37^, Shih-Lung Cheng^6^, Horng-Chyuan Lin^7,8,38^, Jung-Yien Chien^1^, Hao-Chien Wang^1,38^; Taiwan Bronchiectasis Research Collaboration (TBARC)

^1^ Department of Internal Medicine, National Taiwan University Hospital, National Taiwan University College of Medicine, Taipei, Taiwan

^2^ Medical Research Center, Cardinal Tien Hospital and School of Medicine, College of Medicine, Fu Jen Catholic University, New Taipei City, Taiwan

^3^ Department of Internal Medicine, National Taiwan University Hospital Hsin-Chu branch, HsinChu, Taiwan

^4^ Division of Pulmonary and Critical Care Medicine, Department of Internal Medicine, Kaohsiung Medical University Hospital, Kaohsiung, Taiwan

^5^ Department of Internal Medicine, School of Medicine, College of Medicine, Kaohsiung Medical University, Kaohsiung, Taiwan

^6^ Division of Thoracic Medicine, Far Eastern Memorial Hospital, New Taipei City, Taiwan

^7^ Department of Thoracic Medicine, Chang Gung Memorial Hospital, Linkou, Taoyuan, Taiwan

^8^ College of Medicine Chang Gung University, Taoyuan, Taiwan

^9^ Division of Pulmonary and Critical Care Medicine, Department of Internal Medicine, China Medical University Hospital, Taichung, Taiwan

^10^ Critical Medical Center, China Medical University Hospital, Taichung, Taiwan

^11^ School of Medicine, College of Medicine, China Medical University, Taichung, Taiwan

^12^ Division of Pulmonary and Critical Care Medicine, Department of Internal Medicine, Tri-Service General Hospital, National Defense Medical University, Taipei, Taiwan

^13^ Division of Chest Medicine, Department of Internal Medicine, Taichung Veterans General Hospital, Taichung, Taiwan

^14^ School of Medicine for International Students, College of Medicine, I-Shou University, Kaohsiung, Taiwan

^15^ Department of Internal Medicine, E-Da Cancer Hospital, I-Shou University, Kaohsiung, Taiwan

^16^ Division of Pulmonary and Critical Care Medicine, Chiayi Chang Gung Memorial Hospital, Chiayi, Taiwan

^17^ Division of Pulmonary Medicine, Department of Internal Medicine, Taipei Tzu Chi Hospital, Buddhist Tzu Chi Medical Foundation, New Taipei City, Taiwan

^18^ Department of Internal Medicine, Cardinal Tien Hospital and School of Medicine, College of Medicine, Fu Jen Catholic University, New Taipei City, Taiwan

^19^ Division of Pulmonary Medicine, Department of Internal Medicine, Hualien Tzu Chi Hospital, Buddhist Tzu Chi Medical Foundation, Hualien, Taiwan

^20^ School of Medicine, Tzu-Chi University, Hualien, Taiwan

^21^ Division of Pulmonary Medicine, Department of Internal Medicine, Chia-Yi Chrisitian Hospital, Chiayi, Taiwan

^22^ Division of Pulmonary Medicine, Department of Internal Medicine, Chung Shan Medical University Hospital, Taichung, Taiwan

^23^ School of Medicine, Chung Shan Medical University, Taichung, Taiwan

^24^ Department of Internal Medicine, Division of Chest Medicine, Changhua Christian Hospital, Changhua, Taiwan

^25^ Institute of Genomics and Bioinformatics, National Chung Hsing University, Taichung, Taiwan

^26^ Ph.D. Program in Translational Medicine, National Chung Hsing University, Taichung, Taiwan

^27^ Division of Pulmonary & Critical Care Medicine, Department of Internal Medicine, Kaohsiung Chang Gung Memorial Hospital, Kaohsiung, Taiwan

^28^ Department of Respiratory Therapy, Kaohsiung Chang Gung Memorial Hospital, Kaohsiung, Taiwan

^29^ College of Medicine, Chang Gung University, Taoyuan, Taiwan

^30^ Department of Respiratory Therapy, College of Medicine, Kaohsiung Medical University, Kaohsiung, Taiwan

^31^ Department of Internal Medicine, National Taiwan University Hospital, Yunlin Branch, Douliu, Taiwan

^32^ Thoracic Medicine Center, National Taiwan University Hospital, Yunlin Branch, Douliu, Taiwan

^33^ Department of Medicine, Hualien Armed Forces General Hospital, Hualien, Taiwan

^34^ Department of Critical Care Medicine, Taichung Veterans General Hospital, Taichung, Taiwan

^35^ School of Post Baccalaureate Medicine, College of Medicine, National Chung Hsing University, Taichung, Taiwan

^36^ Department of Internal Medicine, E-Da Hospital, I-Shou University, Kaohsiung, Taiwan

^37^ Department of Pulmonary Medicine, Lee's Clinic, Pingtung, Taiwan

^38^ Department of Respiratory Therapy, Chang Gung Memorial Hospital, Linkou, Taoyuan, Taiwan

^39^ Department of Medicine, National Taiwan University Cancer Center, National Taiwan University College of Medicine, Taipei, Taiwan

^†^ **Correspondence:** Cheng-Yi Wang (cywang@mospital.com)

**Contents**

**Supplementary Table 1.** Immunoglobulin E level and eosinophil count in smokers or non-smokers page 5

**Supplementary Table 2.** Results of pulmonary function testing in high-eosinophil and low-eosinophil groups stratified by IgE level page 6

**Supplementary Table 3.** Odds ratio of acute exacerbation requiring hospitalization and (B) all-cause mortality in high-eosinophil and low-eosinophil group page 7

**Supplementary Table 4.** Univariate and multivariate logistic regression analysis for severe acute exacerbation requiring admission in one year after excluding patients administrated with biologics page 8

**Supplementary Figure 1.** Distribution of eosinophil count in respective to IgE level. page 9

**Supplementary Figure 2.** Radiographic patterns. Shown are the distribution of lobar involvement, proportion of tree-in-bud pattern, total lobes involved, and modified Reiff score in (A) high-eosinophil and (B) low-eosinophil cohort stratified by IgE level. page 10

**Supplementary Table 1.** Immunoglobulin E level and eosinophil count in smokers or non-smokers

|  | Smoker (N=171) | Non-smoker (N=408) | *P* |
| --- | --- | --- | --- |
| Immunoglobulin E (IU/mL) | 156.3±301.3 | 168.9±339.8 | 0.67 |
| Eosinophil (/uL) | 189.5±0.49 | 173.0±0.50 | **< 0.001** |

**Supplementary Table 2. Results of pulmonary function testing in high-eosinophil and low-eosinophil groups stratified by IgE level**

|  | Entire cohort (N=480) | High-eosinophil group | | | | | Low-eosinophil group | | | | |
| --- | --- | --- | --- | --- | --- | --- | --- | --- | --- | --- | --- |
|  |  | All  (N=66) | IgE ≤ 100 IU/mL (N=40) | 100< IgE ≤ 500 IU/mL (N=14) | IgE > 500 IU/mL  (N=12) | *P* | All  (N=414) | IgE ≤ 100 IU/mL (N=287) | 100< IgE ≤ 500 IU/mL (N=92) | IgE > 500 IU/mL  (N=35) | *P* |
| FEV_1_ (L) | 1.54±0.65 | 1.61±0.64 | 1.63±0.66 | 1.62±0.54 | 1.51±0.70 | 0.84 | 1.53±0.65 | 1.53±0.64 | 1.51±0.67 | 1.52±0.69 | 0.94 |
| FEV_1_ (%) | 69.6±22.7 | 69.3±23.2 | 70.8±23.7 | 67.9±19.6 | 65.6±26.5 | 0.77 | 69.6±22.7 | 70.3±23.4 | 68.8±19.9 | 66.5±24.1 | 0.60 |
| FVC (L) | 2.15±0.81 | 2.24±0.72 | 2.31±0.77 | 2.23±0.68 | 2.03±0.60 | 0.50 | 2.14±0.83 | 2.14±0.81 | 2.15±0.87 | 2.09±0.86 | 0.93 |
| FVC (%) | 76.6±21.5 | 75.7±21.7 | 79.7±19.9 | 67.5±25.9 | 71.9±20.4 | 0.16 | 76.7±21.5 | 77.1±21.1 | 76.8±22.0 | 73.7±23.7 | 0.68 |
| FEV_1_/FVC | 72.7±13.5 | 71.9±13.5 | 71.2±13.6 | 73.1±11.6 | 72.8±16.1 | 0.88 | 72.8±13.5 | 73.4±13.5 | 71.0±13.6 | 73.1±13.2 | 0.32 |
| TLC (L) | 4.82±1.32 | 5.01±1.22 | 5.32±1.21 | 4.84±1.07 | 4.33±1.21 | 0.23 | 4.78±1.34 | 4.90±1.38 | 4.51±1.28 | 4.56±0.98 | 0.33 |
| TLC (%) | 93.7±21.2 | 96.2±22.2 | 96.8±18.4 | 110.4±32.0 | 82.6±17.4 | 0.11 | 93.1±21.1 | 95.3±20.7 | 87.0±21.9 | 91.9±19.8 | 0.17 |
| DLCO (%) | 96.2±40.5 | 88.4±31.5 | 99.9±12.3 | 74.0±22.6 | 73.5±46.5 | 0.27 | 98.1±42.3 | 100.9±47.7 | 85.5±19.6 | 106.0±7.6 | 0.45 |

FEV_1_, forced expiratory volume in one second; FVC, forced vital capacity; IgE, immunoglobulin E; TLC, total lung capacity; DLCO, diffusion capacity of carbon monoxide

**Supplementary Table 3. Odds ratio of acute exacerbation requiring hospitalization and (B) all-cause mortality in high-eosinophil and low-eosinophil group**

|  | Any acute exacerbation admission in one year | | | All-cause mortality in one year | | |
| --- | --- | --- | --- | --- | --- | --- |
|  | IgE ≤ 100 IU/mL | 100 < IgE ≤ 500 IU/mL | IgE > 500 IU/mL | IgE ≤ 100 IU/mL | 100 < IgE ≤ 500 IU/mL | IgE > 500 IU/mL |
| Entire cohort | 0.699 (0.424-1.151) | 0.716 (0.380-1.347) | 3.203 (1.685,6.087) | 0.661 (0.184-2.371) | 0.916 (0.192-4.369) | 2.486 (0.514-12.01) |
| High-eos cohort | 0.625 (0.215-1.815) | 0.706 (0.215-2.319) | 5.353 (0.658-43.56) | 0.519 (0.069-3.879) | 1.489 (0.145-15.32) | 1.769 (0.171-18.36) |
| Low-eos cohort | 0.679 (0.218-2.111) | 0.462 (0.140-2.939) | 3.592 (0.947-13.62) | 0.856 (0.155-4.727) | 0.710 (0.082-6.145) | 2.297 (0.262-20.16) |

Eos, absolute eosinophil count; IgE, immunoglobulin E

**Supplementary Table 4. Univariate and multivariate logistic regression analysis for severe acute exacerbation requiring admission in one year after excluding patients administrated with biologics**

|  | Univariate analysis | | Multivariate analysis | |
| --- | --- | --- | --- | --- |
|  | Odds ratio | *P* | Odds ratio | *P* |
| Age (age) | 0.992 (0.972-1.011) | 0.40 | 1.071 (1.027-1.117) | 0.001 |
| Male | 1.340 (0.826-2.173) | 0.24 | 0.555 (0.158-1.948) | 0.36 |
| Body mass index (kg/m^2^) | 1.032 (0.972-1.095) | 0.30 | 0.933 (0.829-1.050) | 0.25 |
| Smoking | 1.271 (0.762-2.121) | 0.36 | 0.791 (0.218-2.869) | 0.72 |
| Asthma | 1.311 (0.802-2.142) | 0.28 | 1.230 (0.498-3.037) | 0.65 |
| Chronic obstructive pulmonary disease | 1.750 (1.051-2.914) | **0.03** | 1.993 (0.735-5.410) | 0.18 |
| ICS/LABA | 1.504 (0.903-2.505) | 0.12 | 2.371 (0.960-5.854) | 0.06 |
| Absolute eosinophil count (/uL) | 1.017 (1.013-1.021) | **< 0.001** | 1.021 (1.016-1.027) | **< 0.001** |
| Immunoglobulin E > 500 IU/mL | 3.341 (1.721-6.488) | **< 0.001** | 7.158 (2.243-22.85) | **0.001** |

ICS, inhaled corticosteroid; LABA, long-acting beta-adrenoceptor agonists

**Supplementary Figure 1.** Distribution of eosinophil count in respective to IgE level. IgE, immunoglobulin E

**
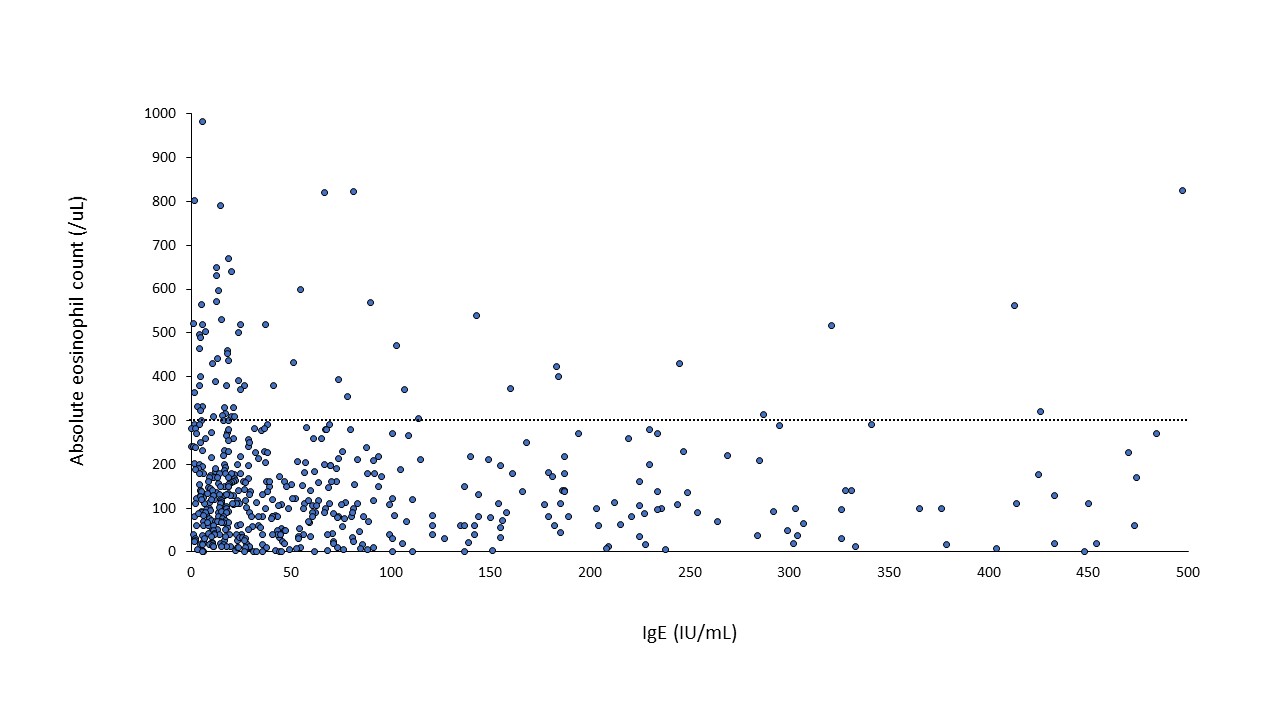
**

**Supplementary Figure 2. Radiographic patterns.** Shown are the distribution of lobar involvement, proportion of tree-in-bud pattern, total lobes involved, and modified Reiff score in (A) high-eosinophil and (B) low-eosinophil cohort stratified by IgE level. IgE, immunoglobulin E


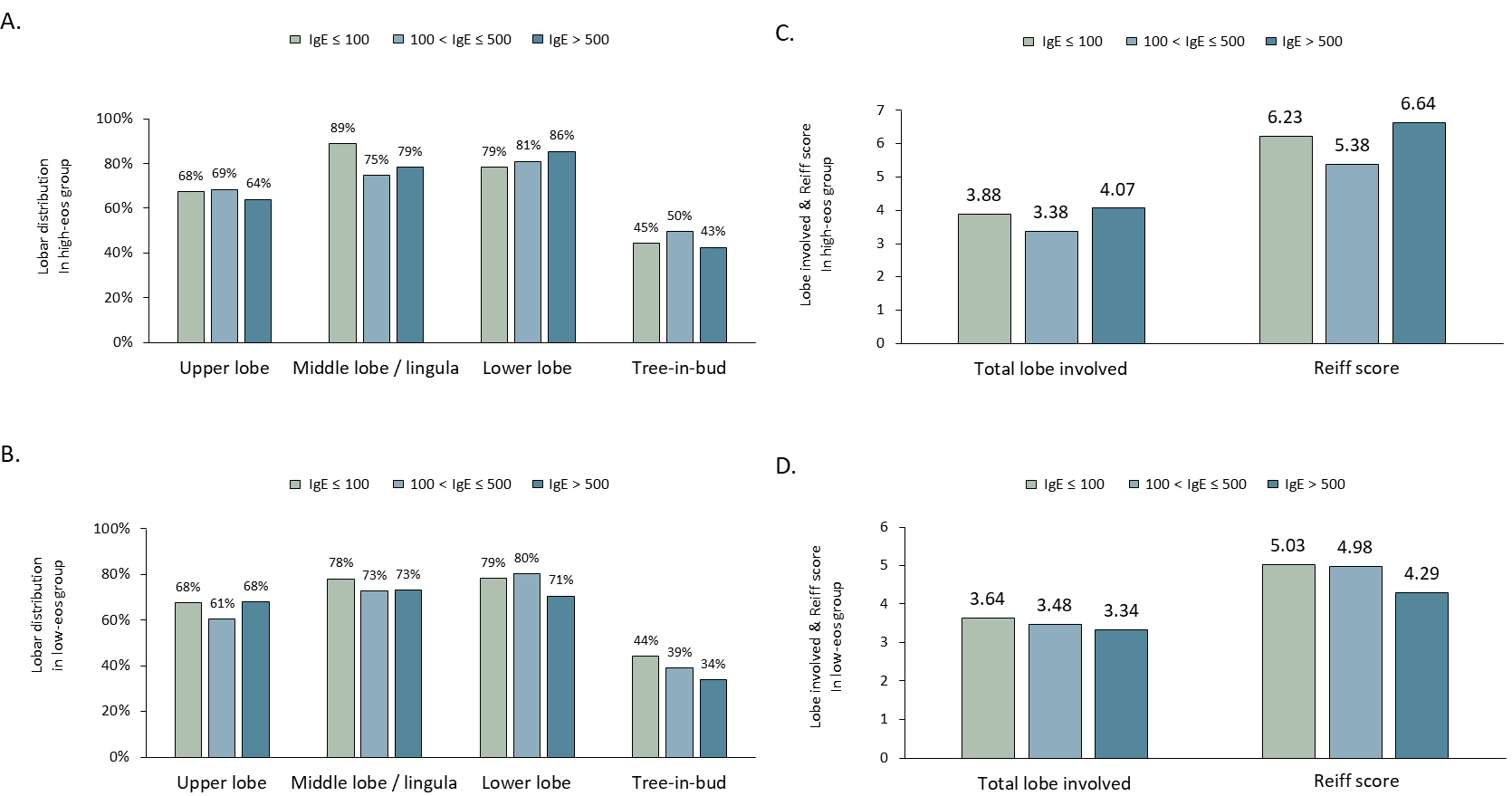

Supplement: Supplementary file 1 — (DOCX 19 kb) [file 408_2026_874_MOESM1_ESM.docx]
